# The influence of surgical technique guidance and surgeon’s experience on the femoral head assembly in total hip arthroplasty

**DOI:** 10.1007/s00402-024-05282-w

**Published:** 2024-04-02

**Authors:** Martin Darowski, Leo Ruehrmund, Daniel Kluess, Annett Klinder, Rainer Bader, Wolfram Mittelmeier

**Affiliations:** Department of Orthopaedics, Rostock University Medical Center, Doberaner Straße 142, D-18057, Rostock, Germany

**Keywords:** Total hip arthroplasty, Taper connection, Femoral head, Assembly procedure, Surgical technique guide, Morse taper

## Abstract

**Introduction:**

The importance of the assembly procedure on the taper connection strength is evident. However, existent surgical technique guides frequently lack comprehensive and precise instructions in this regard. The aim of our experimental study was to evaluate the influence of the surgical technique guide on the femoral head assembly procedure in surgeons with differing levels of experience in total hip arthroplasty.

**Materials and methods:**

Twenty-eight participants, divided into four groups based on their lifetime experience in total hip arthroplasty, conducted a femoral head assembly procedure in a simulated intraoperative environment before and after reviewing the surgical technique guide. Demographic information and the number of hammer blows were documented. Hammer velocity and impaction angle were recorded using an optical motion capturing system, while the impaction force was measured using a dynamic force sensor within the impactor.

**Results:**

We observed a high variation in the number of hammer blows, maximum force, and impaction angle. Overall, the number of hammer blows decreased significantly from 3 to 2.2 after reviewing the surgical technique guide. The only significant intragroup difference in the number of hammer blows was observed in the group with no prior experience in total hip arthroplasty. No correlation was found between individual factors (age, weight, height) or experience and the measured parameters (velocity, maximum force and angle).

**Conclusions:**

The present study demonstrated a high variation in the parameters of the femoral head assembly procedure. Consideration of the surgical technique guide was found to be a limited factor among participants with varying levels of experience in total hip arthroplasty. These findings underline the importance of sufficient preoperative training, to standardize the assembly procedure, including impaction force, angle, and use of instruments.

**Supplementary Information:**

The online version contains supplementary material available at 10.1007/s00402-024-05282-w

## Introduction

The concept of modularity at the head-neck junction in primary total hip arthroplasty (THA) offers surgeons the flexibility to adjust size and offset of the femoral head, neck and stem to the anatomy of the patient. However, modularity provides the femoral component with an additional interface, which is a potential source of wear and corrosion.

Taper wear and corrosion involve a complex interplay of many factors that mostly depend on implant design and surgical technique. Retrieval studies indicate that more rigid trunnions and tapers with a smaller taper angle are less susceptible to corrosion and fretting [1–4]. In recent decades there has been a trend towards smaller trunnions as they have the inherent advantage of a bigger head-neck-ratio with an increased impingement-free range of motion. Despite their clinical advantages, smaller trunnions are less rigid and may result in a higher risk of taper corrosion [2].

The underlying process of significant tribocorrosion in the junction between the metallic stem taper and the femoral head is referred to as mechanically assisted crevice corrosion (MACC) and can result in the release of metal corrosion products into the joint fluid. These particles and metal ions can lead to an adverse local tissue reaction (ALTR) with osteolysis and cytotoxic effects (necrosis) in the surrounding bone stock and soft tissues [5, 6]. While this problem is well known in metal-on-metal bearings, studies showed a prevalence of MACC from 1.1 to 3.2% in metal-on-polyethylene bearings for total hip arthroplasties [7, 8], which are still a common bearing in total hip arthroplasty up to date [9, 10]. A recent clinical study showed a revision rate of 11.6% for MACC with metal-on-polyethylene bearing with a mean time to revision of 6.6 years [11]. Although ALTR is less common with ceramic femoral heads, there are some reports about ALTR with ceramic-on-polyethylene and ceramic-on-ceramic bearings [12–14]. In addition to the design of the total hip implant, there is increasing evidence that the assembly procedure of modular components plays a crucial role for taper stability and thus for fretting and corrosion of modular hip endoprostheses [15–17]. Reduced wear in the taper-head junction with increasing assembly forces was reported [18, 19].

Experimental studies advised an assembly of the femoral head with a force of at least 4 kN with one hammer blow with the femoral head placed on a clean, dry taper [20–22]. However, preclinical studies face challenges in simulating the intraoperative setting during the femoral head assembly and often neglect the influence of the human factor.

Although the method for femoral head impaction is described and illustrated in the surgical technique guide, information differs about the number of hammer blows, impaction force, and impaction angle depending on the used implant [23]. Therefore, the purpose of our experimental study was to evaluate the effect of the surgical technique guide on variables of the femoral head assembly procedure (number of hammer blows, hammer velocity, maximum impaction force and impaction angle) of participants with different experience in THA.

## Materials and methods

### Experimental test rig

**Fig. 1:**
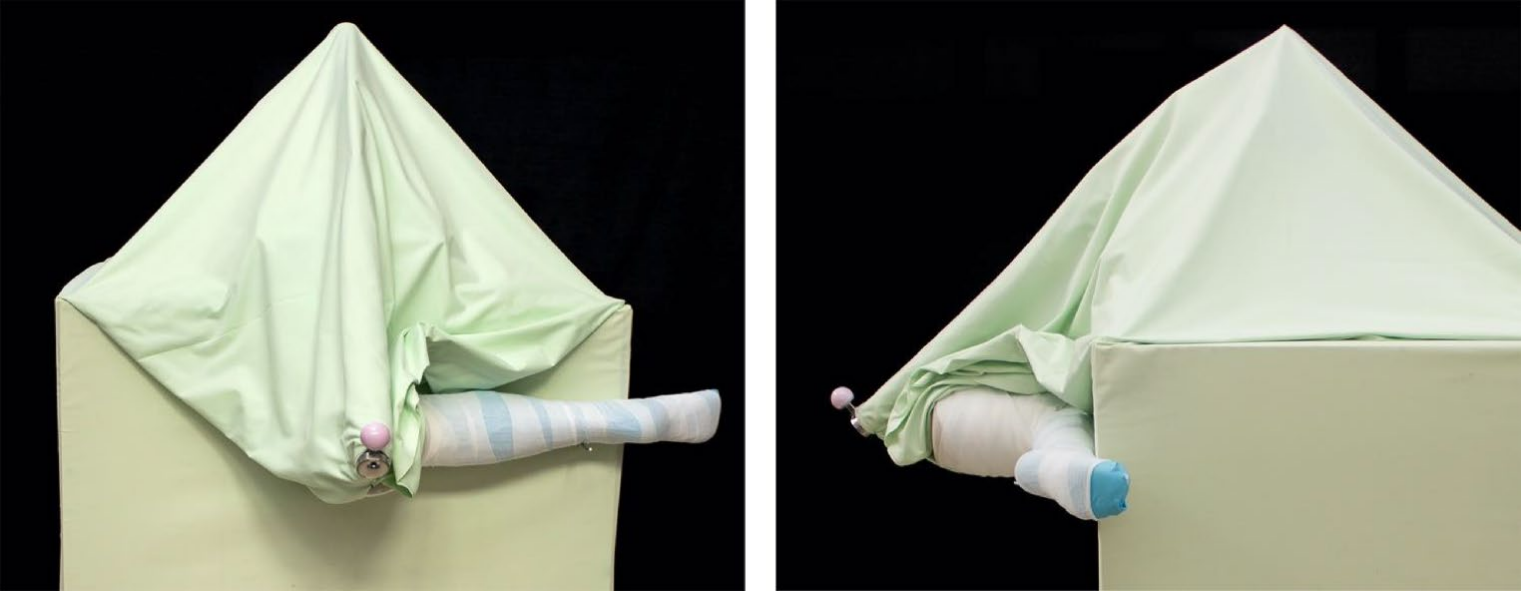
Test rig with artificial left leg to simulate a patient lying in supine position with cover of the spring and the aluminium profile construction

An experimental test rig to simulate intraoperative conditions during a femoral head assembly procedure was developed (Fig. 1). A cementless stem (LCU, Size 11, 12/14 taper, Ti6Al4V4, Waldemar Link GmbH & Co. KG, Hamburg, Germany) was embedded in a polyurethane casting (Rencast FC 52 Isocyanate / FC 52 Polyol, Huntsman Advanced Materials; Salt Lake City, UT, USA) in an iron alloy pipe (made of X6CrNiMoTi17-12-2) that acted as a spring to simulate the elastic behaviour of a leg. The pipe had an analytically calculated stiffness of 275 N/mm in the longitudinal taper axis, which was in agreement to spring stiffness in previous studies [24]. We fixed the pipe to an aluminium profile construction (item Industrietechnik GmbH, Solingen, Germany) which was mounted on a wooden base. To simulate a patient lying in supine position with an antero-lateral hip approach on the left side we connected an artificial leg to the stem and covered the test rig with large surgical drapes (Fig. 1). For the impaction trials a BIOLOX®delta ceramic head with a diameter of 36 mm (CeramTec GmbH, Plochingen, Germany) was placed on the taper.

### Hammer and impactor

**Fig. 2:**
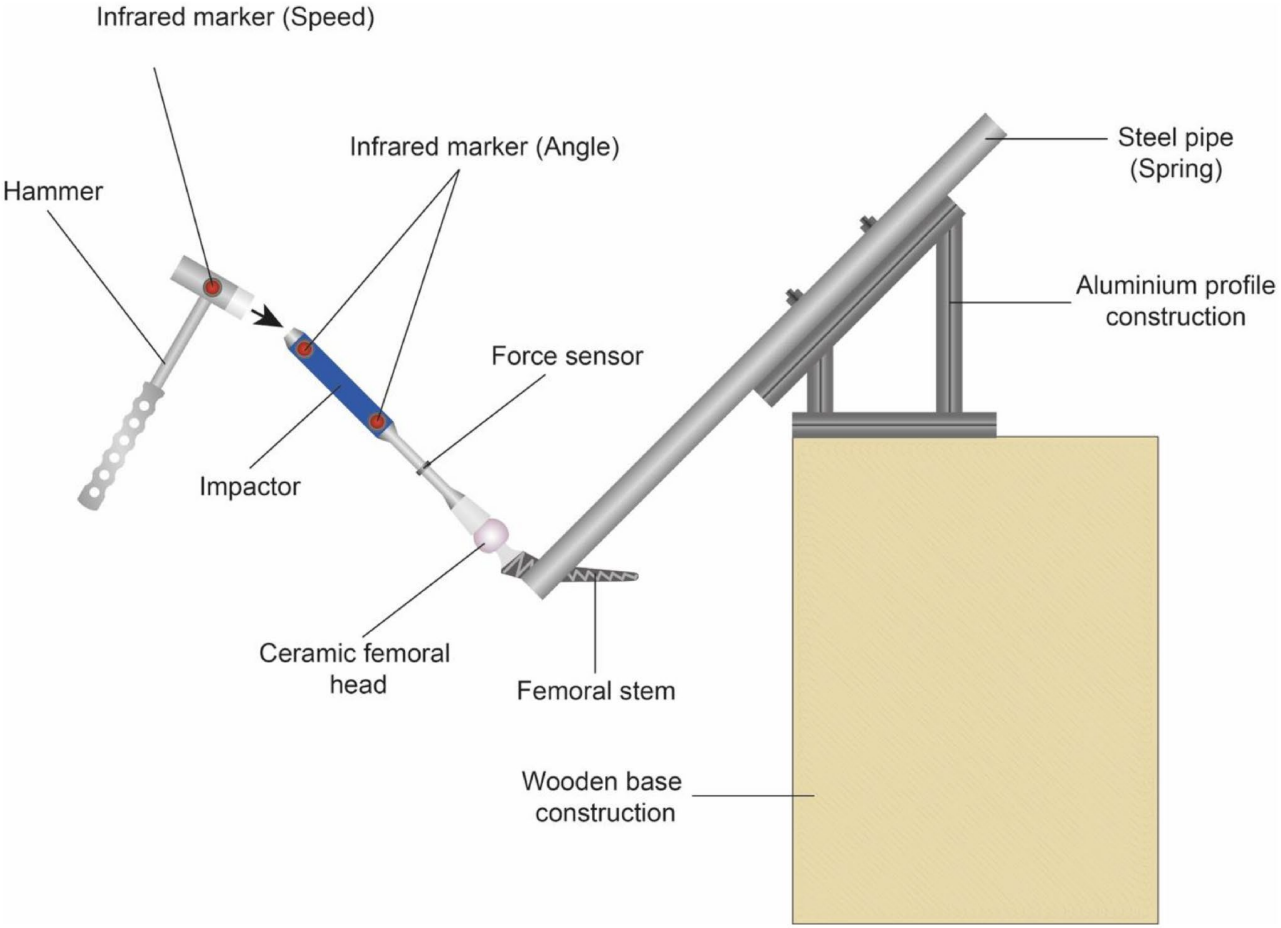
Schematic representation of the test rig without cover

We used a commercially available impaction hammer (mass = 758 g, Waldemar Link GmbH & Co. KG, Hamburg, Germany) with two impaction sides: stainless steel or polyamide (PA12). For our trials we used only the stainless-steel impaction side of the hammer. We also used a commercially available impactor consisting of a stainless-steel impact area and a PA12 tip (mass = 416 g, Waldemar Link GmbH & Co. KG, Hamburg, Germany). We modified the shaft of the impactor to mount a force sensor along the shaft axis (Fig. 2). This was realized by sawing the shaft into two at its centre and turning threads into the single shaft parts to fix the sensor.

### Force measurement

The force measurement was performed with a dynamic force sensor (Model M201A76, PCB Piezotronics, Depew, USA) with a capacity of 22 kN, which was supplied with energy by a piezotron coupler (Type 5134B, Kistler Instrumente GmbH, Sindelfingen, Germany). The output voltage of the piezotron coupler was sampled with 100 kHz and 16 bit by an analog to digital converter (Model USB 6216, National Instruments, Austin, TX, USA) resulting in a resolution of 0.73 N.

Additionally, the force sensor was manually calibrated before the tests. This was performed using a static testing machine (Model Z50, ZwickRoell GmbH & Co. KG, Ulm, Germany) equipped with a 50 kN load cell (GTM Testing and Metrology GmbH, Bickenbach, Germany) by means of static loading (duration > 120 s) and as fast as possible relieving (duration < 1 s). The force amplitude shown at the relieving process was evaluated as the measured force of the sensor. This process was performed at multiple force values (500–5,000 N). The coefficient of determination of the correction curve was > 0.9999.

Before calibration and testing, an initial compression pre-load of at least 4.5 kN was applied to the force sensor according to the instructions given by the manufacturer. The analysis of the force data was performed with LabView Version 13 (National Instruments, Austin, TX, USA). The absolute force was evaluated by subtracting the signal before impact from the signal peak caused by the impact. The error range for the force measurements was ± 10%.

### Angle and velocity measurement

The angle and velocity were recorded with an infrared optical tracking system (Model MCU200, LUKOtronic, Innsbruck, Austria), with the corresponding software. Three infrared markers were used (upper impactor, lower impactor, and hammer) which were sampled at 240 Hz. The impaction angle measurement was performed by calculating the linear curve between the two infrared markers mounted at the impactor (Fig. 2). Before starting the tests, the zero angle was determined by positioning the impactor on a socket that was connected to the femoral stem equal to the taper axis. The angle that was measured during the tests was the solid angle in relation to the zero angle. The error range for the angle measurement was ± 3°.

For the velocity measurement, the coordinate of the hammer marker was captured. This marker was positioned in a way that captured the absolute velocity of the hammer head without axial rotation. A software computed function was used to differentiate the velocity data from the motion data.

Trajectories, velocity, and force data were collected and evaluated with a script using MATLAB 2018a (MathWorks, Natick, MA, USA). In this script the absolute velocity of the hammer-marker, equivalent to the velocity of the hammer head, was calculated based on the assumption, that the hammer releases the entire kinetic energy (at some point the velocity equals zero) at the end of the impaction. The angles were calculated for every hammer blow at the time point when the velocity was at the maximum. The error range for the velocity measurement was ± 5%.

### Impaction trials

A total of 28 participants, consisting of 17 orthopaedic surgeons with different experience in THA and 11 non-surgeons took part in the study. We divided the participants into four study groups according to their lifetime experience in THA. Group 1 had no experience in THA, Group 2 performed 1–10 THA, Group 3 performed 11–100 THA and Group 4 performed > 100 THA during their career. We documented the age, height, and weight of the participants. The experimental setup was split into two sections. In the first section, the participants were asked to hit the femoral head according to their experience or, for the unexperienced participants, according to their idea how to hit the femoral head, respectively. The impactor, that was placed on the femoral head was hit with the metal side of the hammer. The participants had three attempts to perform the assembly procedure. In the second section, the participants were provided with the available surgical technique guide of the THA implant system for the assembly procedure with following instructions: “Clean and dry the taper of the stem thoroughly. This is particularly important with ceramic heads. Mount the head by hand using axial pressure and a turning motion. Impact the head lightly if necessary, using the impactor for prosthesis heads”. After reading the surgical technique guide, the assembly procedure was carried out again according to the first part of the experimental setup. The femoral head was disassembled before each attempt and was placed on the taper by the participants. To optimise the position of the subject during the assembly procedure, we offered a step stool to account for different body heights.

### Statistics

The differences between the four groups for maximum force, hammer velocity, impaction angle and variance were evaluated with Kruskal-Wallis test and Dunn´s as the post hoc test. Non-parametric tests were chosen as analysis with Shapiro-Wilk test revealed that the data were not normally distributed. Maximum force, hammer velocity, impaction angle and variance and overall differences within a group before and after reading the surgical technique guide were compared statistically using Wilcoxon test for a paired, non-parametric analysis. Correlations between maximum force, impaction angle, hammer velocity, height, weight, age, and group were calculated using the Spearman rank test. Cut-off for positive or negative correlation was r_s_< -0.5 or r_s_> 0.5.

The analysis was performed using Graph Pad Prism 8 (GraphPad Software, San Diego, CA, USA) with a significance level of 0.05. All results are reported as mean ± standard deviation (range).

## Results

Twenty-eight participants with a mean age of 37.4 years ± 11.3 years (20 years – 68 years), a mean height of 178.2 cm ± 9.4 cm (155 – 198 cm) and a mean weight of 78.5 kg ± 16.9 kg (53 – 115 kg) participated in the study. All participants were right-handed. Detailed data for the single groups are presented in Table 1.

**Table 1:** Personal data of the participants in the four study groups

Group	1	2	3	4
n	11	4	9	4
Age (y)	31.6 ± 10.7 y	31.3 ± 1.3 y	40.2 ± 4.1 y	52.3 ± 12.3 y
Height (cm)	176.4 ± 7.1 cm	176.3 ± 16.8 cm	181.1 ± 7.8 cm	179.8 ± 11.1 cm
Weight (kg)	74.9 ± 10.9 kg	71.3 ± 17.2 kg	85.7 ± 16.8 kg	81.3 ± 21.1 kg

### Number of hammer blows

A total of 438 hammer blows from 28 participants with 6 attempts each (3 attempts before and 3 attempts after reading the surgical technique guide) were recorded, 255 before and 183 after reading the surgical technique guide. The mean number of hammer blows before reading the surgical technique guide over all participants and attempts was 3.0 ± 1.7 (1–11), which decreased significantly to 2.2 ± 1.1 (1–5) after reading the surgical technique guide (*p* < 0.0001).

The average number of hammer blows per attempt for the four separate groups is shown in Table 2. Group 2 used significantly lower numbers of hammer blows on the femoral head for assembly than Group 3 before reading the surgical technique guide (*p* < 0.05). After reading the surgical technique guide there were no significant differences between the groups.

Within the groups there was a significant decrease in number of hammer blows in Group 1 after reading the surgical technique guide (*p* < 0.05). In Groups 2, 3 and 4 reading the surgical technique guide had no significant influence on the number of hammer blows.

**Table 2:** Number of hammer blows in the four study groups. The data show the values of three attempts each before and after reading the surgical technique guide

Group		1		2		3		4	
Surgical technique guide		Before	After	Before	After	Before	After	Before	After
Number of hammer blows	Minimum	1	1	1	1	2	1	1	1
	Median	3	2	1	1	4	3	1.5	1.5
	Maximum	11	4	3	3	5	5	5	3
	Mean	3.2	2.0	1.5	1.5	3.9	2.9	2.3	1.75
	SD	1.6	1.0	1.0	1.0	1.2	1.1	1.9	1.0

SD (standard deviation)

### Influence of surgical technique guide on hammer velocity, maximum force and impaction angle

The mean data for hammer velocity, maximum force and impaction angle are presented in Table 3.

**Table 3:** Data for hammer velocity, maximum force and impaction angle acquired in three attempts each before and after reading the surgical technique guide in the four study groups

Group		1		2		3		4	
Surgical technique guide		Before	After	Before	After	Before	After	Before	After
Velocity (m/s)	Min.	1.455	1.084	1.738	1.329	0.522	0.577	1.229	1.174
	Median	3.136	2.696	3.185	2.670	1.753	1.162	2.769	2.207
	Max.	4.538	5.710	3.974	4.077	3.119	2.117	4.844	5.735
	Mean	3.003	3.010	3.020	2.687	1.611	1.216	2.903	2.831
	SD	0.949	1.466	0.937	1.561	0.824	0.518	1.671	2.027
Max. Force (N)	Min.	3,445	2,231	5,174	2,979	1,546	1,128	3,072	3,141
	Median	8,361	7,114	7,669	7,869	6,395	3,017	7,185	5,730
	Max.	20,269	22,828	8,483	11,774	10,973	8,138	16,230	14,346
	Mean	8,666	8,234	7,249	7,623	5,677	3,712	8,418	7,237
	SD	4,582	5,735	1,563	4,717	3,361	2,210	6,123	5,248
Angle (°)	Min.	3.8	2.4	4.1	2.3	3.5	1.8	3.9	6.3
	Median	8.2	7.5	6.9	6.0	5.6	5.3	7.3	8.5
	Max.	29.9	17.0	9.9	7.3	26.0	20.1	24.3	34.6
	Mean	11.3	8.2	6.9	5.4	10.7	8.0	10.7	14.5
	SD	7.8	4.3	2.7	2.3	8.6	6.7	9.2	13.5

SD (standard deviation)

#### Hammer velocity

The average hammer velocity calculated from all hammer blows was 2.544 ± 1.171 m/s (0.522 m/s – 4.844 m/s) before and 2.362 ± 1.503 m/s (0.577 m/s – 5.735 m/s) after reading the surgical technique guide without a significant change (*p* = 0.227).

**Fig. 3:**
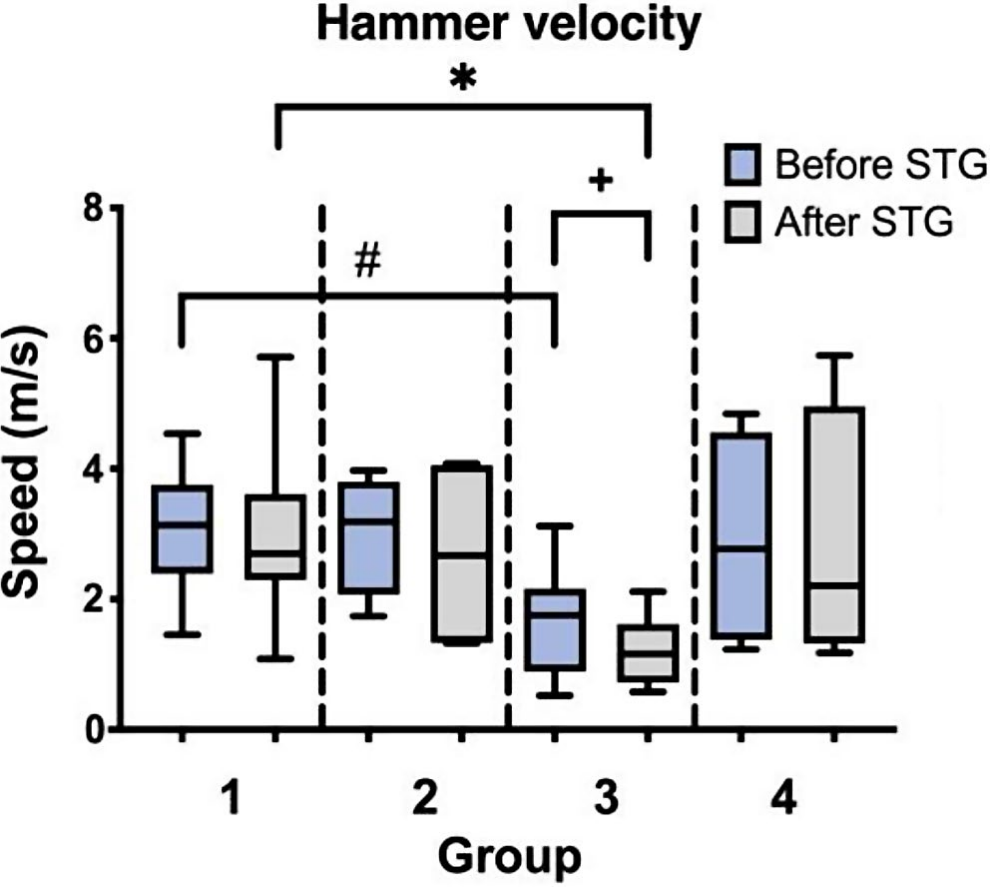
Hammer velocity by Group before and after reading the surgical technique guide (STG). The hammer velocity of Group 3 was significantly lower than of Group 1 (* *p* = 0.0225) after reading the surgical technique guide (STG) and showed a similar trend (# *p* = 0.0558) before reading the surgical technique guide (STG). Comparison of intragroup difference showed a non-significant trend towards a decreasing hammer velocity in Group 3 after reading the surgical technique guide (STG) (+ *p* = 0.0742)

Hammer velocity differed between Group 1 and Group 3 with lower hammer velocities recorded in Group 3. While before reading the surgical technique guide this was only a trend (*p* = 0.0558) the difference between the two groups reached significance after reading the surgical technique guide (*p* = 0.0225). In general, Group 3 showed a non-significant trend towards decreased velocity after reading the surgical technique guide (*p* = 0.0742) (Fig. 3).

#### Maximum Force

**Fig. 4:**
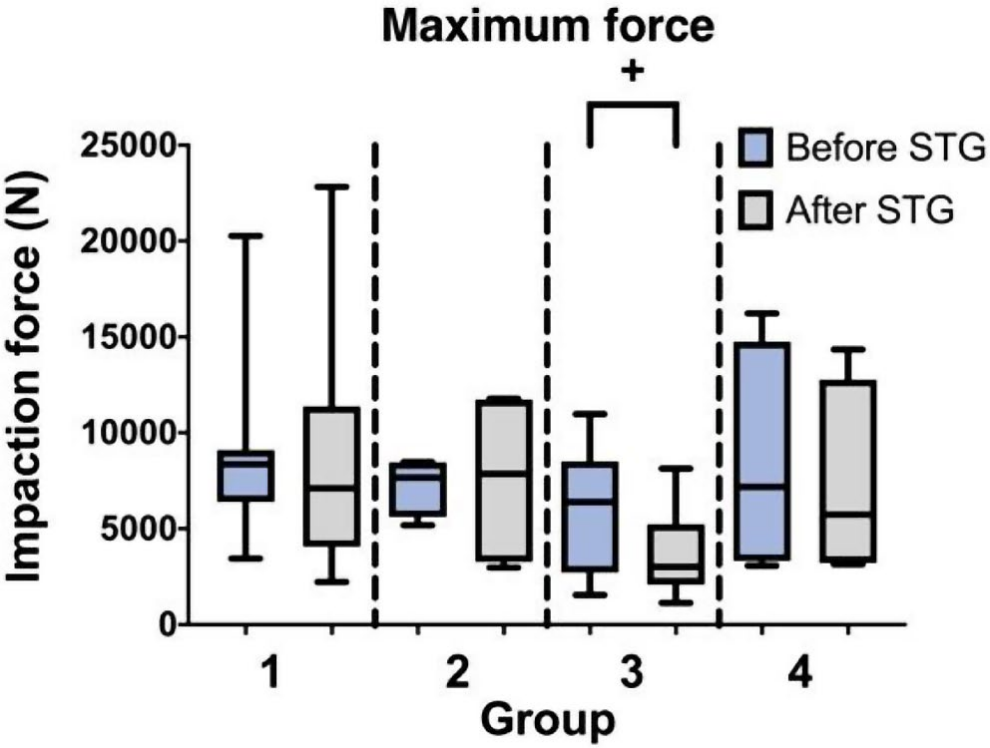
Maximum impaction force within the four study groups before and after reading the surgical technique guide (STG). The maximum impaction force of Group 3 showed a trend (+ *p* = 0.0558) towards decreased values after reading the surgical technique guide (STG)

The overall average maximum force before reading the surgical technique guide was 7,467 *N* ± 4,166 N (1,546 N – 20,269 N) and did not significantly change after reading the surgical technique guide with 6,551 *N* ± 4,821 N (1,128–22,828 N). There was no significant difference between the groups in the maximum applied force (Fig. 4). However, there was a non-significant trend towards a decreased maximum force in Group 3 (*p* = 0.0558) after reading the surgical technique guide.

#### Impaction angle

**Fig. 5:**
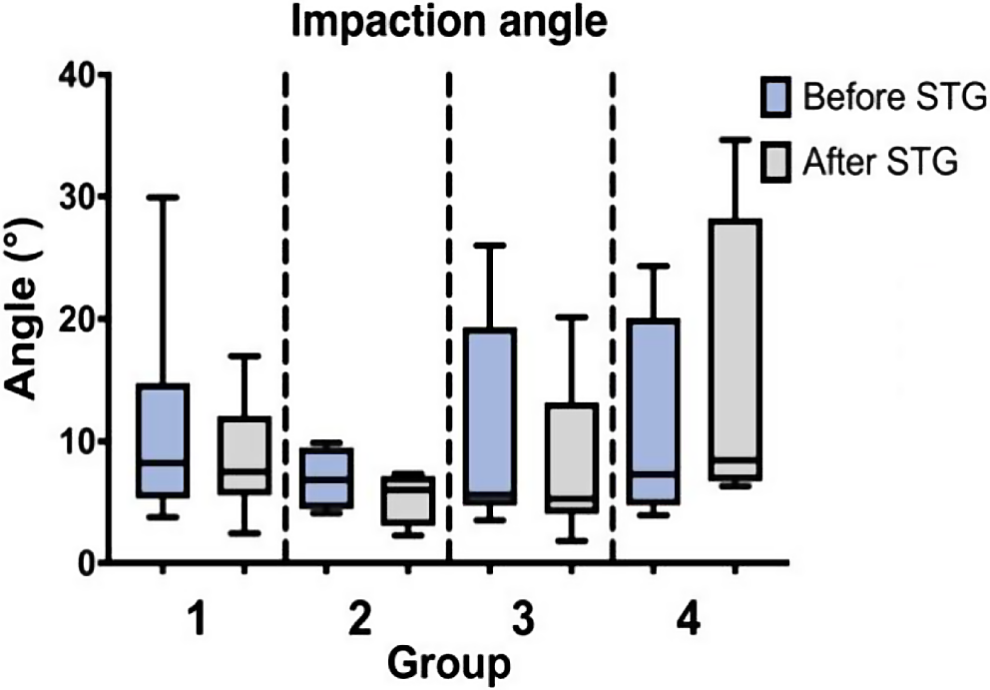
Impaction angle within the four study groups before and after reading the surgical technique guide (STG)

For all hammer blows the average impaction angle was 10.4° ± 7.5° (3.5° – 29.9°) before and 8.7° ± 6.9° (1.8° – 34.6°) after reading the surgical technique guide. There were no significant changes in impaction angle between the groups and no significant change after reading the surgical technique guide (Fig. 5).

### Intergroup and intragroup variance in impaction parameters

**Fig. 6:**
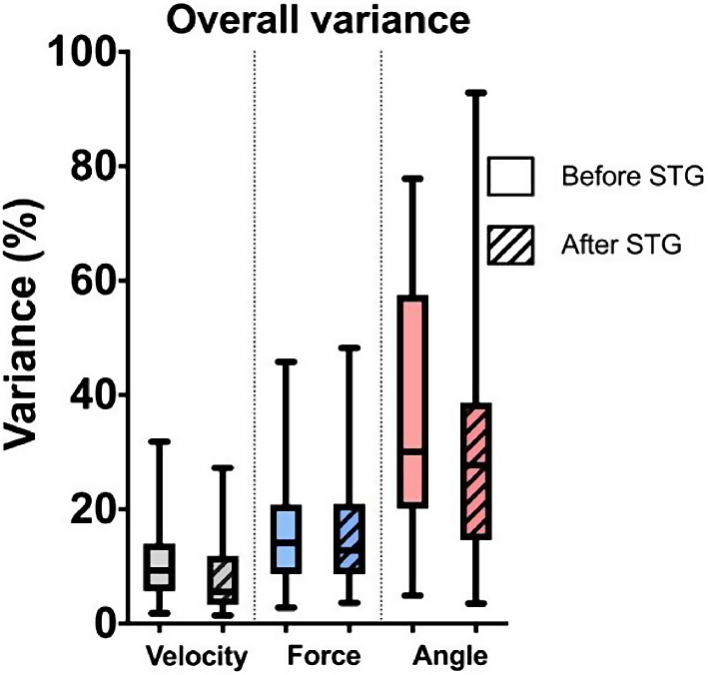
Overall variance of hammer velocity, maximum impaction force and angle before and after reading the surgical technique guide (STG)

The angle, at which the femoral head was hit, varied widely between participants with coefficients of variation (CV) of 36.8% ± 22.5% (4.9 – 77.8%) and 30.4% ± 21.9% (3.5 – 92.9%) before and after reading the surgical technique guide, respectively. While there was also a high variation for the applied force (CV before reading the surgical technique guide: 16.1% ± 10.23% (2.8 − 45.8%); CV after reading the surgical technique guide: 15.9% ± 9.1% (3.6 − 48.2%)), the velocity of the hammer was relatively homogenous (CV before reading the surgical technique guide: 11.1% ± 7.2% (1.8 − 31.8%); CV after reading the surgical technique guide: 8.4% ± 7.0% (1.5 − 27.2%)). There was no significant difference between variance of impaction angle, force, and hammer velocity before and after reading the surgical technique guide (Fig. 6). Also, no significant intergroup or intragroup differences were found in the coefficient of variance of impaction angle, maximum force, and hammer velocity.

### Correlations

There was no correlation between experience in THA and number of hammer blows. In the personal data, there was a positive correlation between experience and age (*p* < 0.001, r_s_= 0.65) as well as between height and weight (*p* < 0.0001, r_s_=0.79). No correlations between personal data (age, height, weight) and measured variables (number of hammer blows, maximum force, hammer velocity, impaction angle) were observed. Hammer velocity and maximum impaction force showed a positive correlation before (*p* < 0.0001, r_s_=0.89) and after reading the surgical technique guide (*p* < 0.0001, r_s_=0.94). The impaction angle showed no correlation to any other measured variables.

## Discussion

The aim of this study was to examine the impact of the surgical technique guide on the femoral head assembly procedure among participants with different levels of experience in THA. While the number of hammer blows was not explicitly stated, reading the surgical technique guide resulted in a significant overall decrease in the number of hammer blows used during the procedure. However, this effect was only significant in Group 1 (no experience in THA). Moreover, reading the surgical technique guide did not have any significant intragroup impact on the hammer speed, force, and angle during the assembly procedure. Additionally, it was observed that force and impaction angle exhibited a high variation among the participants. Overall, the consideration of the surgical technique guide demonstrated only a limited effect on the assembly procedure, highlighting the need for sufficient preoperative training and corresponding instruments to achieve standardized head assembly.

### Number of hammer blows

The assembly procedure plays a pivotal role in establishing the initial stability of the taper connection to prevent excessive micromotion, which may lead to corrosion. Despite its fundamental importance in the assembly procedure, there is still no consensus about the proper surgical technique. The information provided by most surgical technique guides lacks specific information about the instruments that should be used for impaction and about the parameters of impaction (number of hammer blows, impaction force, impaction angle). The assembly procedure for the femoral head shows even conflicting recommendations across various surgical technique guides [23]. Recent preclinical studies give contradictory recommendations concerning the number of hammer blows during the femoral head assembly procedure. Rehmer et al. [20] and Pandorf et al. [25] advise a single hammer impact according to their finding that additional impactions did not increase the pull-off force. The same was confirmed by Pennock et al. [22] who reported that the first impaction accounted for 90% of the mechanical strength of the taper connection. In contrast, Heiney et al. [26] suggested to apply at least two hammer blows as they found a significant increase in pull-off force after the second, third or fourth blow in comparison to hitting the head once. In the present study the mean number of hammer blows before reading the surgical technique guide was three, which was in accordance to previous studies [27, 28]. Although, no exact number of hammer blows was specified in the surgical technique guide, the information provided resulted in a decrease of the overall number of blows. When analysing the intragroup difference, this was only significant in Group 1 (no experience in THA). The number of blows in Group 2–4 decreased after reading the surgical technique guide but did not reach significance. This could be due to the already lower numbers of blows in these groups before reading the surgical technique guide (maximum 5 blows in Group 2–4 compared to 11 in Group 1) as well as the limited number of participants. It is noteworthy, that there was no significant difference in number of blows between non-surgeons and surgeons before and after surgical technique guide.

### Force

Numerous studies have demonstrated a positive correlation between the assembly force and the initial stability of the taper connection [20, 22, 29, 30]. In the current study, the utilized surgical technique guide recommended to “Mount the head by hand using axial pressure and a turning motion” and to “Impact the head lightly if necessary, using the impactor for prosthesis heads”. The terms “axial pressure” “turning motion” and “impact lightly” provide the user with a broad range of interpretations resulting in substantial variations in the assembly procedure among different surgeons. The strongest impaction force in the present study after reading the surgical technique guide (22,828 N) was 20-fold higher than the weakest impaction (1,128 N) which shows how differently the information given in the surgical technique guide regarding the femoral head assembly procedure is interpreted. The impaction forces for the femoral head assembly recorded in our study (1,128 N – 22,828 N) are consistent with forces in other studies that ranged between 273 N and 26,602 N [26, 27, 31, 32].

However, there was no significant intra- and intergroup difference in the maximum force before or after reading the surgical technique guide. These findings are in contrast to Brial et al. [28] who found a positive correlation between years in practice and the average maximum impaction force for attending surgeons. The recommended assembly force depends on head size and is indicated with 4,000 N to 6,000 N in trials that were carried out under laboratory conditions [20, 33]. This force range was met in our study by 22% of the applied impactions before and by 18% after reading the surgical technique guide. The rest of the impactions was below or above the indicated range. On the one hand there is a risk to produce an insufficient taper connection with the consequence of higher wear and corrosion rates as recent studies demonstrated for inferior assembly forces [18, 34]. On the other hand, excessive forces can cause damage to ceramic implant components and adjacent bone tissue [18, 35, 36].

The measured force depends on several conditions, like the location of force acquisition, the material of the hammer, impactor, and the compliance of the system. Lavernia et al. who reported the lowest average forces of 1,661 N ± 148 N, used a force transducer on which eight surgeons performed impactions with an impactor and a hammer [31]. Nassutt et al. [32] used an experimental setup with a force sensor placed under a CoCr taper on a socket with a rubber mat to simulate the intraoperative compliance. The reported minimal force of 273 N ± 24 N was approximately four times lower than our minimal force and corresponded to the maximal force that they reported for assembly of the head by a turning motion with the hand (204 N ± 21 N) [32]. Heiney et al. [26] reported mean impaction forces for attending orthopaedic surgeons to be 4,409 N ± 660 N and 4,346 N ± 939 N for resident orthopaedic surgeons using a pressure sensitive film without specifying the exact assembly conditions. Scholl et al. used a bench-top-model with a stem fixed in a sawbone femur and a force sensor located in the tip of a metal hammer. With this setup, which was similar to our setup, they reported impaction forces with a range from 3,597 N to 26,602 N [27]. However, a study examining the impaction force applied by surgeons conducted by Wendler et al. [37] with human cadavers and damping simulating human soft tissue showed considerably lower impaction forces (822.5 N to a maximum of 3835.2 N) than our study.

### Impaction angle and hammer velocity

Factors, that have so far received little attention, are the impaction angle and hammer velocity. The hammer velocity showed a highly positive correlation with the maximum impaction force acting as an indicator for the impaction force.

Up to date there are only few investigations analysing the impaction angle [38] and there is no literature known to the authors that deals with the influence of the impaction angle on the taper connection strength. However, a study examining factors influencing the femoral head seating displacement could identify impaction angle and assembly force as the most significant factors for impaction assembly [39]. Studies carried out under laboratory conditions use a drop hammer that applies a reproducible axial force. Furthermore, the Standard Test Method for Determining the Axial Disassembly Force of Taper Connections of Modular Prostheses, stipulates a quasi-axial load application, “so that the line of load application is aligned with the axes of the male and female taper components within ± 1°” [40]. However, our results indicate that an axial force application is not usual under realistic conditions. Furthermore, in our study the angle showed the highest variance among the impaction parameters with no significant change after reading the surgical technique guide.

### Clinical implications

The variation of assembly force and angle among surgeons with different experience in total hip arthroplasty in our study persisted, even after advice from the surgical technique guide was provided. The fact demonstrates that there is still a huge potential for a more standardized femoral head assembly process. From the various factors that can positively affect the stability of the taper connection, the impaction force was pointed out as the most important factor in a recent study [17]. The femoral head assembly process during surgery can be influenced by the surgeon. Proper assembly ensures an effective locking of the head-taper connection and reduces potential micromotion. While experimental setups like in this study provide surgeons with perfect conditions for the assembly, the situation in situ usually presents further hurdles for a standardized femoral head assembly. Depending on the anatomy of the patients, the surgical approach, and the design of the used instruments, the assembly process can be negatively influenced, resulting in an unfavourable angle and transduced force of the femoral head impaction. This means an insufficient interlocking of the head-taper connection can lead to MACC and ALTR [6]. With revision rates up to 11.6% for MACC in non-metal-on-metal bearings [11, 14], the problem of MACC is still one of the common severe complications in total hip arthroplasty. A femoral head assembly procedure with instruments that standardize impaction force and angle could be a potential key factor to reduce MACC and consecutive complications as ALTR.

### Limitations

The results of the specific instrumentation system are not generally transferable to other surgical systems. Additionally, our developed test rig represents a more realistic but still artificial test system as no soft tissue that could distract the impactor was implemented. Furthermore, the resulting forces below the taper and disassembly forces or moments were not measured in the present study.

### Conclusion

Consideration of the surgical technique guide had only limited influence on the femoral head assembly procedure of surgeons with varying levels of experience in total hip arthroplasty who participated in the present study. These findings underline the importance of sufficient preoperative training to standardize the assembly procedure, including impaction force, angle, and use of instruments. Otherwise, the fixation strength of the taper connection and consecutively the taper corrosion may vary. The data emphasize the need for precise personal instructions including the definition of the number of hammer blows, impaction force, impaction angle and impaction instruments. Moreover, our results indicate that laboratory test setups should represent more realistic conditions of the intraoperative assembly procedure of the femoral head.

### Electronic supplementary material

Below is the link to the electronic supplementary material.

Supplementary Material 1

Supplementary Material 2

Supplementary Material 3

Supplementary Material 4

Supplementary Material 5

Supplementary Material 6
